# Butyrate Ameliorates Mitochondrial Respiratory Capacity of The Motor-Neuron-like Cell Line NSC34-G93A, a Cellular Model for ALS

**DOI:** 10.3390/biom12020333

**Published:** 2022-02-19

**Authors:** Xuejun Li, Li Dong, Ang Li, Jianxun Yi, Marco Brotto, Jingsong Zhou

**Affiliations:** 1 Department of Kinesiology, College of Nursing and Health Innovation, University of Texas at Arlington, Arlington, TX 76019, USA; xuejun.li@uta.edu (X.L.); li.dong@uta.edu (L.D.); ang.li3@uta.edu (A.L.); jianxun.yi@uta.edu (J.Y.); 2 Bone-Muscle Research Center, College of Nursing and Health Innovation, University of Texas at Arlington, Arlington, TX 76019, USA; marco.brotto@uta.edu

**Keywords:** butyrate, PGC1α, mitochondria, NSC34 cell line, ALS

## Abstract

Mitochondrial defects in motor neurons are pathological hallmarks of ALS, a neuromuscular disease with no effective treatment. Studies have shown that butyrate, a natural gut-bacteria product, alleviates the disease progression of ALS mice overexpressing a human ALS-associated mutation, hSOD1^G93A^. In the current study, we examined the potential molecular mechanisms underlying the effect of butyrate on mitochondrial function in cultured motor-neuron-like NSC34 with overexpression of hSOD1^G93A^ (NSC34-G93A). The live cell confocal imaging study demonstrated that 1mM butyrate in the culture medium improved the mitochondrial network with reduced fragmentation in NSC34-G93A cells. Seahorse analysis revealed that NSC34-G93A cells treated with butyrate showed an increase of ~5-fold in mitochondrial Spare Respiratory Capacity with elevated Maximal Respiration. The time-dependent changes in the mRNA level of PGC1α, a master regulator of mitochondrial biogenesis, revealed a burst induction with an early increase (~5-fold) at 4 h, a peak at 24 h (~19-fold), and maintenance at 48 h (8-fold) post-treatment. In line with the transcriptional induction of PGC1α, both the mRNA and protein levels of the key molecules (MTCO1, MTCO2, and COX4) related to the mitochondrial electron transport chain were increased following the butyrate treatment. Our data indicate that activation of the PGC1α signaling axis could be one of the molecular mechanisms underlying the beneficial effects of butyrate treatment in improving mitochondrial bioenergetics in NSC34-G93A cells.

## 1. Introduction

Amyotrophic lateral sclerosis (ALS) is a neuromuscular disease characterized by progressive motor neuron loss and severe skeletal muscle wasting. Two FDA-approved therapies (Riluzole and Radicava) only extend the life span by a few months, while the progressive skeletal muscle paralysis in ALS usually affects respiratory function, frequently leading to ventilatory failure and death in 3–5 years following the disease diagnosis. Thus, various research efforts have been made to develop potential therapeutic means for battling ALS. Among those, butyrate has shown promising outcomes in alleviating disease progression in an ALS mouse model overexpressing the human ALS mutation SOD1^G93A^ (G93A) [1,2]. However, the underlying molecular mechanisms remain to be further explored.

Most cases of ALS are sporadic (SALS), with about 10% being familial (FALS). While diverse factors contribute to sporadic and familial ALS pathogenesis, there are commonly shared pathological and clinical features in all ALS cases, suggesting that different initiating causes lead to a mechanistically similar neurodegenerative pathway [3,4]. As a main power supply resource, mitochondria are vital organelles for neuronal function since the brain consumes 20% of the body’s resting production of ATP, while it makes up only 2% of the body’s mass [5]. Accumulating evidence indicates mitochondrial dysfunction as a major contributor to ALS pathology [6,7]. Spinal cord and muscle autopsy/biopsy samples derived from both sporadic and familial ALS patients show remarkable mitochondrial defects, i.e., morphological/ultrastructural abnormalities and impaired oxidative metabolism [8,9,10,11,12,13], indicating abnormal mitochondria as a common player in neuromuscular degeneration in ALS patients [14,15,16]. Mouse models expressing human ALS mutations (e.g., SOD1^G93A^) recapitulate many features of the human disease [17] and have been widely used to investigate pathogenic mechanisms and preclinical therapies [7,14,18,19,20]. Studies of ALS transgenic models have shown that accumulation of mutant SOD1 proteins inside mitochondria likely contributes to the dysfunction of motor neurons [21,22]. Transgenic mice with SOD1^G93A^ targeted to mitochondria develop ALS symptoms [23,24,25,26,27]. These mice also show impaired mitochondrial bioenergetics and a reduced number of spinal motor neurons [23]. Additionally, mutant SOD1 has been found located in the brain mitochondrial matrix, while SOD1^G93A^ aggregates in transgenic mice [25]. Mutant SOD1 in transgenic mice altered the morphology and distribution of axonal mitochondria and affected axonal transport [26,28]. Our studies using the ALS G93A mouse model also established a role for mitochondrial Ca^2+^ and ROS signaling in mediating the crosstalk between muscle and neurons in ALS [29,30,31,32,33]. We showed that segmented mitochondrial dysfunction took place in proximity to neuromuscular junctions prior to symptom onset [31,33], further supporting mitochondrial damage as a factor contributing to neuromuscular degeneration in ALS [34].

Butyrate, a short-chain fatty acid produced by bacterial fermentation of dietary fibers in the colon, is essential for gut homeostasis [35,36]. As gut-microbe-derived metabolites enter circulation, butyrate has been found to influence the physiology of many organs [37,38,39]. Apparently, butyrate can cross the blood–brain barrier and exhibit neuroprotective effects in various neurodegenerative diseases [40]. The neuroprotective effects of butyrate have been associated with its role as an inhibitor of histone deacetylase (HDAC) [41]. As inhibition of HDAC could impact the transcription of numerous genes, multiple signaling pathways should be involved in its neuroprotective effects. Our recent study showed that butyrate feeding reversed the ALS mutation hSOD1^G93A^-induced mitochondrial ROS signaling in skeletal muscle myofibers, but the involved upstream signaling pathways remain elusive [42].

Peroxisome proliferator-activated receptor-r coactivator 1α (PGC1α) plays a central role in the regulation of mitochondrial metabolism and biogenesis [43,44,45]. A reduced mRNA level of PGC1α was reported in both human sporadic ALS and an ALS G93A mouse model [46]. A study by Zhao et al. found that neuronal-specific overexpression of PGC1α significantly improved the motor function and survival of ALS G93A mice [47], although a later study from the same group reported that systemic overexpression of PGC1α did not confer a significant improvement in the G93A mice [48]. It is likely that systemic overexpression could lead to other complications. In fact, studies from other research groups have linked the salutary effect of butyrate in mitochondrial function and the biogenesis of muscle and neuronal cells via PGC1α activation [49,50]. In the current study, we used the cultured motor-neuron-like cell line NSC34 with overexpression of the mitochondrial-targeted human ALS-associated mutation hSOD1^G93A^ (termed NSC34-G93A cells). Our goal was to examine how butyrate treatment improves mitochondrial function in a more specific way (i.e., in motor-neuron-like cells) and to dissect the time-dependent response of PGC1α and its downstream key molecules related to mitochondrial bioenergetics at both the transcriptional and translational levels in NSC34-G93A cells.

## 2. Materials and Methods

### 2.1. NSC34 Cells and Culture Conditions

NSC34 mouse motor neuron enriched hybrid cells were purchased from Cellutions Biosystems Inc., Canada. Cells were routinely cultured in DMEM containing 5% Fetal Bovine Serum at 37 °C, 5% CO_2_. Transfection was carried out with Lipofectamine 3000 regent (Thermo Fisher, L3000008, Dallas, TX, USA) according to the manufacturer’s manual. The plasmid pcDNA-mt-SOD1^G93A^-GFP, which overexpresses mitochondrial target human SOD1^G93A^ mutant protein, was described previously [51].

### 2.2. Life Imaging of Cultured Cells

NSC34 cells were seeded on glass-bottom dishes coated with poly-D-lycine and ECM. Cells were transfected with pcDNA-mt-SOD1G93A-GFP plasmid using Lipofectamine 3000 reagent according to the manufacturer’s manual. After overnight incubation, cells were treated with 1mM butyrate for a specific time, and live cell imaging was recorded under a TC SP8 confocal microscope (Leica, Chicago, IL, USA).

### 2.3. Seahorse Cell Mito Stress Analysis

XF DMEM medium, XF24 culture plates, XF24 FluxPak, and a Cell Mito Stress kit were purchased from Agilent (Santa Clara, CA, USA). NSC34 cells were seeded in the XF24 plates at 5~10 × 10^3^ cells per well, transfected with plasmid pcDNA-mt-SOD1^G93A^-GFP, and then treated with 1 mM butyrate, as described in the text. The oxygen consumption rate (OCR) of cultured cells was determined with an Agilent Seahorse XFe24 Analyzer, following the manufacturer’s instructions. After the assay, the cells were immediately stained with Hoechst33324 (ENZO, New York, NY, USA), staining the nucleus). The whole wells of stained cells were imaged through the tile scan method under a Leica DMi8 microscope. The stitched images were imported into ImageJ for background subtraction, thresholding, binarization, and cell counting.

### 2.4. RNA Extraction and qPCR

The total RNA was extracted from cultured cells using the Direct-zol RNA miniprep plus kit from Zymo Research (Irvine, CA, USA). The RNA concentration was measured using a Quantus Fluorometer (Promega, Madison, WI, USA), and reverse transcription was carried out using the Promega GoScript Reverse Transcription system. Maxima SYBR Green/ROX qPCR Master Mix (Thermo Fisher, Dallas, TX, USA) was used for qPCR reactions. The qPCR primers were ordered from Sigma (Saint Louis, MO, USA). The sequences of the primers are listed in Table 1. qPCR was carried out using the StepOnePlus Real-Time PCR system (Thermo Fisher). Comparative C_T_ (ΔΔC_T_) assessment was used to quantify the relative mRNA level with reference to β-actin.

### 2.5. Analysis of the mtDNA/nDNA Ratio

The ratio of mitochondrial DNA (mtDNA) to nuclear DNA (nDNA) was determined via qPCR (Quiros 2017 with modifications). The total DNA was isolated from cultured NSC34 cells using the QuickDNA MiniPrep kit from Zymo Research (Zymo Research, D3024, Irvine, CA, USA), and qPCR was carried out as described above. The primers for mitochondrial gene ND2 and nuclear gene HK2 are also listed in Table 1.

### 2.6. Immunoblotting Assay

The protein was extracted from cultured NSC34-G93A cells using Laemmli SDS loading buffer and heated for 8 min at 90 °C. Protein samples were resolved via SDS-polyacrylamide gel electrophoresis (SDS-PAGE), then transferred to a PVDF membrane with a Bio-Rad semidry transfer cell (1703940, Plano, TX, USA). Membranes were blocked with 5% milk in TBS with 0.1% Tween-20 for 1 h. The primary antibodies used in this study were PGC1α (Abcam ab110324), MT-CO1 (CST 62101S), MT-CO2 (Proteintech 55070-1-AP), COX4 (CST 4844S), and Actin (CST 4970S). After overnight incubation with primary antibody at 4 °C, the membranes were washed with TBST buffer and then incubated with horseradish-peroxidase-conjugated secondary antibody at room temperate for 2 h. Protein bands were visualized using a Bio-Rad Clarity ECL kit under the ChemiDoc Imaging System (Bio-Rad Laboratory). The band intensity was analyzed using ImageJ software.

### 2.7. Statistical Analysis

Data are expressed as the mean ± SE (standard error). Differences between two groups were analyzed via a two-sided unpaired Student’s t-test. The distribution of cells with different contents of networked mitochondria was analyzed via Pearson’s chi-square test. A *p*-value of 0.05 or less was considered to indicate statistical significance. Box-and-dot plots were generated with the ggplot2 package of R.

## 3. Results

### 3.1. Butyrate Treatment Improved the Mitochondrial Network in NSC34-G93A Cells

**Figure 1:**
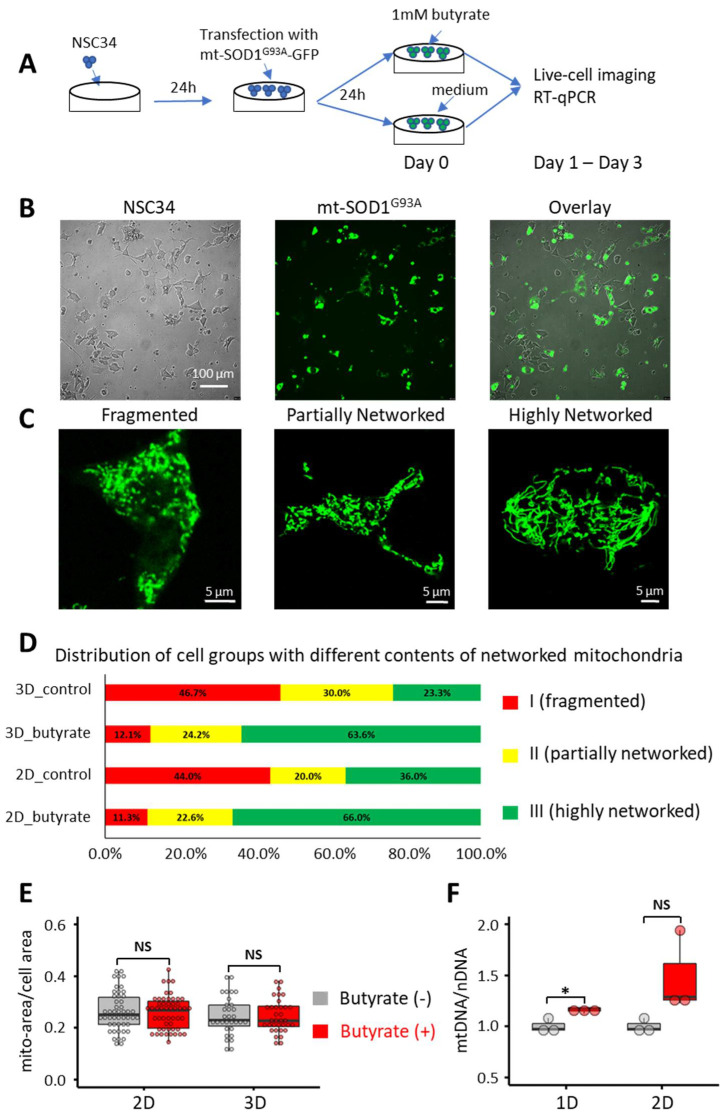
Butyrate incubation improved the mitochondrial network of NSC34-G93A cells. (**A**) A schematic illustration of the experimental procedure. (**B**) Representative images of NSC34 cells transfected with pcDNA-mt-SOD1^G93A^GFP. The GFP fluorescence identifies NSC34-G93A cells. (**C**) Representative images of NSC34-G93A cells with **Fragmented**, **Partially Networked**, and **Highly Networked** mitochondria. A length/diameter ratio greater than 5 was defined as indicating networked mitochondria. (**D**) Distribution of cell populations with different contents of networked mitochondria with or without 1 mM butyrate in the culture medium for 2 or 3 days (2D or 3D). Note that butyrate incubation increased the percentage of cells with highly networked mitochondria. (**E**) Mitochondria volume was estimated with the ratio of mitochondrial area (mito-area) to the whole cell area (cell area) (*n* = 30/group). (**F**) The ratio of mitochondrial DNA to nuclear DNA (mtDNA/nDNA) quantified by RT-qPCR (*n* = 3). For the box and dot plot, the box bottom, median line, and box top represent the 25th (Q1), 50th (Q2), and 75th (Q3) percentiles, respectively. Whisker ends represent Q1 − 1.5*IQR and Q3 + 1.5*IQR, respectively. IQR is the interquartile range (Q3 − Q1). * *p* < 0.05; NS: not significant.

The NSC34 motor-neuron-like cell culture has been used by various research groups as an in vitro cellular model of ALS to study the effect of ALS-associated mutations on motor neuron function [52,53]. In previous studies, we also examined whether the ALS-associated mutation hSOD1^G93A^ had a direct contribution to mitochondrial dysfunction in both muscle and bone osteocyte MLO-Y4 cells by overexpressing mitochondrion-targeted hSOD1^G93A^ (mt-SOD1^G93A^-GFP) [51,54]. Here, we used the same method to generate an ALS motor neuron cellular model by overexpressing mt-SOD1^G93A^-GFP in NSC34 cells. As shown in Figure 1B, the NSC34 cells were transfected with mt-SOD1^G93A^-GFP, and the GFP fluorescence allowed us to identify the cells with overexpression of hSOD1^G93A^, which we named NSC34-G93A cells for this study. As illustrated in Figure 1A, to limit the potential influence of the variable degree of cell differentiation, the butyrate effects were compared between the dishes passed from the same mother culture, which received the same transfection and were examined under confocal microscopy on the same day. The following qRT-PCR and immunoblotting assessments followed the same experimental design.

We first examined whether butyrate treatment affected the mitochondrial network in the cultured NSC34-G93A cells. After the transfection with mt-SOD1^G93A^-GFP, the cultured cells were incubated with or without 1 mM sodium butyrate (butyrate), and the morphology of mitochondria was examined using live cell confocal microscope imaging for 2 or 3 days after the butyrate incubation. The concentration of 1 mM was chosen based on published studies. The butyrate concentration in human circulation is in the micromolar range [55,56]. Thus, the butyrate treatment in the millimolar concentration range could have therapeutic potential. The dose-dependent effects of butyrate on diverse in vitro cellular models have been tested in the millimolar range [57,58,59], in which the 1 mM concentration has been tested the most [60,61,62,63,64].

To quantify the morphological change to mitochondria in NSC34-G93A cells, we classified the cells into three groups based on the contents of networked mitochondria. A networked mitochondrion was defined as one with a length/diameter ratio larger than 5. The cells with fragmented mitochondria (Fragmented) are those that contain less than 30% networked mitochondria. The cells with partially networked mitochondria (Partially Networked) are those that contain 30–50% networked mitochondria, and the cells with highly networked mitochondria (Highly Networked) are those that contain more than 50% networked mitochondria. Representative images of each cell group are presented in Figure 1C. Figure 1D shows the distribution of those three cell groups in culture conditions in the presence or absence of the butyrate incubation. The percentage of cells with highly interconnected (Highly Networked) mitochondria increased from 36.0% to 66.0% after 2 days of butyrate incubation (*p* = 0.000642, Pearson Chi-square association test, cell number *n* = 103) and increased from 23.3% to 63.3% after 3 days of butyrate incubation (*n* = 63, *p* = 0.001931, Pearson Chi-square test). There were no significant differences between the data collected at 2 days and 3 days, with or without the butyrate incubation. The Pearson’s chi-square tests showed *p* = 0.4103 when comparing the distributions between 2 days and 3 days for the non-treated groups, with *p* = 0.9745 analogously for the treated groups. These data suggest that butyrate incubation improved the mitochondrial networks in NSC34-G93A cells.

We next examined whether the improved mitochondrial network reflected an increase in total mitochondrial volume. By examining the ratio of the mitochondrion occupied area to the total cell area, we found no significant changes before and after the butyrate treatment, suggesting that the total mitochondrial volume was not significantly changed by the butyrate incubation up to 3 days (Figure 1E), although more networked mitochondria were formed. We also evaluated the ratio of mitochondrial DNA (mtDNA) to nuclear DNA (nDNA) by qPCR. As shown in Figure 1F, we observed a significant 16% increase (*p* = 0.024) in mtDNA relative to nDNA 1 day after 1 mM butyrate incubation. The averaged relative mtDNA level also revealed a 45% increase, although it was not statistically significant (*p* = 0.068) due to one sample having a drastic elevation in the ratio of mtDNA to nDNA.

### 3.2. Butyrate Treatment Improved Mitochondrial Respiration Function of NSC34-G93A Cells

**Figure 2:**
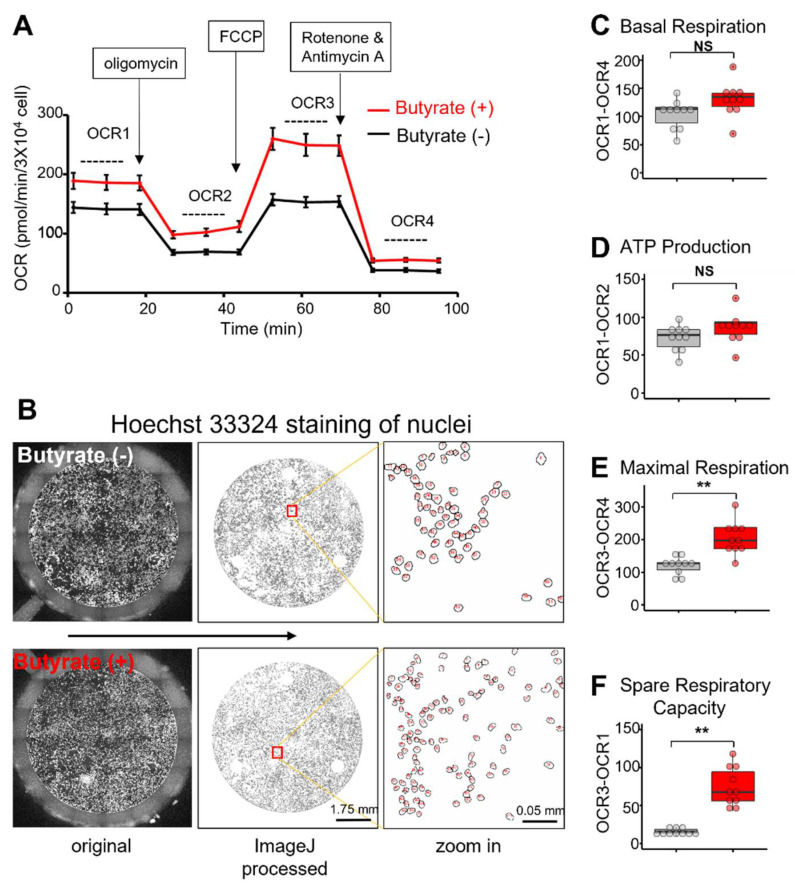
Butyrate treatment improved mitochondrial respiratory function. (**A**) Seahorse XFe24 Cell Mito Stress test: NSC34 cells were first transfected with mt-SOD1^G93A^-GFP. At 24 h after the transfection, the cells were incubated with 1 mM butyrate for 48 h before the measurement of the OCR. Oligomycin, FCCP, and rotenone and antimycin were then injected in succession to enable the measurements of ATP production, maximal respiration, and non-mitochondrial respiration. (**B**) The total cell number in each well was counted by Hoechst 33324 staining after the assay, which was used to normalize the OCR reading. The enlarged areas (right planes) with high resolution demonstrate that individual cells could be counted accurately with ImageJ. (**C**,**D**) There was no significant difference in basal respiration or ATP production in the resting state. (**E**,**F**) The maximal respiration and spare respiratory capacity were significantly increased following the butyrate treatment. For the box and dot plot, the box bottom, median line, and box top represent the 25th (Q1), 50th (Q2), and 75th (Q3) percentiles, respectively. Whisker ends represent Q1−1.5*IQR and Q3 + 1.5*IQR, respectively. IQR is the interquartile range (Q3−Q1). *n* = 10, ** *p* < 0.01; NS: not significant.

To further examine the effect of butyrate on mitochondrial function, we evaluated the mitochondrial respiration by determining the cellular oxygen consumption rate (OCR) in NSC34-G93A cells using a Seahorse XFe 24 Analyzer (Agilent). First, ~10 × 10^3^ cells were seeded per well. One day after the transfection with mt-SOD1^G93A^-GFP, 1 mM butyrate was applied to the culture medium for an additional 48 h of incubation. The control group received no butyrate treatment. Cells were washed twice in pre-warmed serum-free XF assay medium, then incubated in a 37 ℃ non-CO_2_ incubator for 1 h before the measurement. For the Cell Mito Stress test, oligomycin (2 μM), FCCP (1–2 μM), and rotenone and antimycin A (0.5 μM) were sequentially administered, and the OCR was then recorded. After the test, the cells were immediately incubated with Hoechst 33,324 (0.25 μg/mL) for 30 min at 37 ℃ to stain the nuclei. Hoechst-33324-stained cells in each well were imaged via a tile-scanning approach using a Leica epifluorescence microscope (Figure 2B, left panels). The total cell number of each well was counted using ImageJ (Figure 2B, center and right panels) and used to normalize the OCR reading (Figure 2A).

As described in the Agilent Seahorse instructions, oligomycin was used as a complex V (ATP synthase) inhibitor. FCCP is an uncoupling agent that collapses the proton gradient and disrupts the mitochondrial membrane potential. As a result, the electron flow through the ETC is fully activated, and oxygen is maximally consumed by complex IV. The combination of rotenone, a complex I inhibitor, and antimycin A, a complex III inhibitor, shuts down mitochondrial respiration and enables the calculation of non-mitochondrial respiration driven by processes outside the mitochondria. Based on the normalized OCR reading (Figure 1A), basal respiration was calculated by subtracting the non-mitochondrial OCR4 from OCR1 before the first oligomycin injection (OCR1-OCR4) (Figure 2C). ATP production was determined by the difference in OCRs before and after the oligomycin injection (OCR1-OCR2) (Figure 2D). Maximal respiration was determined by the difference in OCRs before and after the rotenone and antimycin A injection (OCR3-OCR4) (Figure 2E). The spare respiratory capacity was determined by subtracting the basal respiration from the maximal respiration (OCR3-OCR1) (Figure 2F). We found that the 48 h of 1 mM butyrate treatment did not significantly alter the basal respiration and ATP production. However, the maximal respiration and, particularly, the spare respiratory capacity were significantly enhanced after the 48 h butyrate treatment.

### 3.3. Butyrate Treatment Induced the Transcription of PGC1α in NSC34-G93A Cells

**Figure 3:**
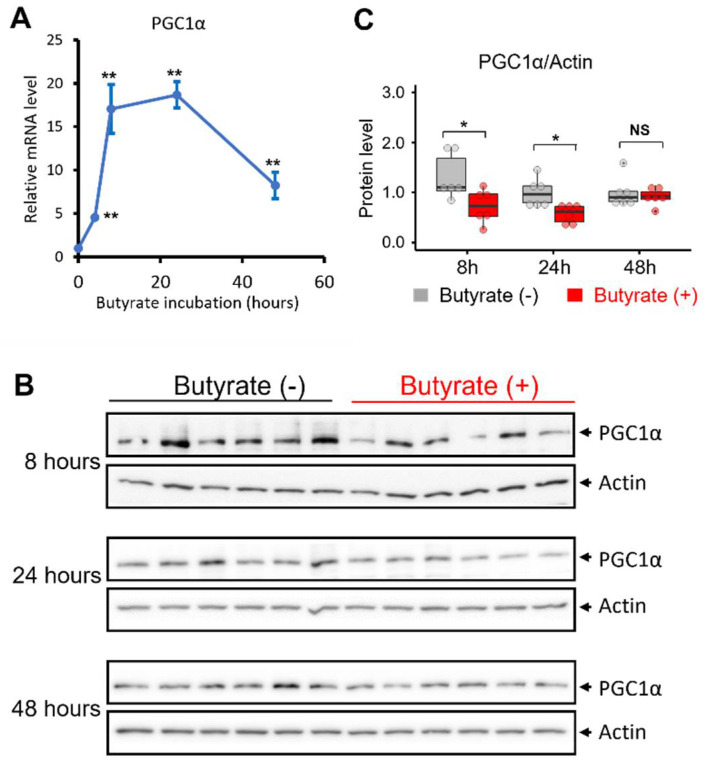
Butyrate treatment induced the transcription of PGC1α. (**A**) A time course of the mRNA level of PGC1α in NSC34-G93A cells was followed during the 1 mM butyrate incubation. Control groups received no butyrate treatment. The relative mRNA level was calculated by treated/non-treated values (*n* = 3 independent experiments). (**B**) Immunoblotting assessment of the PGC1α protein expression level at 8, 24, and 48 h post-treatment. The left six lanes (Butyrate (−)) were samples of the 6 control groups (without butyrate treatment), while the right six lanes (Butyrate (+)) were samples of the six treated groups. (**C**) The PGC1α protein levels were analyzed with ImageJ using actin as a reference protein (*n* = 6 independent experiments). For the box and dot plot, the box bottom, median line, and box top represent the 25th (Q1), 50th (Q2), and 75th (Q3) percentiles, respectively. Whisker ends represent Q1−1.5*IQR and Q3 + 1.5*IQR, respectively. IQR is the interquartile range (Q3−Q1). * *p* < 0.05; ** *p* < 0.01; NS: not significant.

We next investigated the potential molecular basis underlying the effects of butyrate treatment on mitochondrial oxidative phosphorylation. PGC1α is at the center of the regulation of mitochondrial biogenesis [43,44,45]. We examined whether butyrate incubation altered the transcription level of PGC1α in NSC34-G93A cells. NSC34 cells were transfected with mt-SOD1^G93A^-GFP. The incubation of 1 mM butyrate was started 24 h after the transfection. The control groups received no butyrate treatment. By qPCR analysis, we evaluated the mRNA levels of PGC1α in a time window from 4 h to 48 h following 1 mM butyrate incubation. As shown in Figure 3A, a burst in the mRNA level of PGC1α was observed following the butyrate treatment. Four hours after the butyrate treatment, the mRNA level of PGC1α had already increased 4.5-fold and quickly reached 17.1-fold in another 4 h. The mRNA level reached its peak (~19-fold) at 24 h and was maintained as high as 8.2-fold after 48 h of treatment.

We then performed an immunoblotting assay to determine whether the protein level of PGC1α would reflect the transcriptional induction following the butyrate treatment at the three time points of 8, 24, and 48 h, when the mRNA level showed a drastic increase in responding to the butyrate treatment (Figure 3B). Interestingly, while the protein expression level of PGC1α showed no significant change at 48 h after the treatment, it was significantly decreased at 8 and 24 h post-treatment (Figure 3C). This apparent paradoxical result prompted us to further explore the time-dependent changes in PGC1α protein expression at earlier time windows during the butyrate incubation.

**Figure 4:**
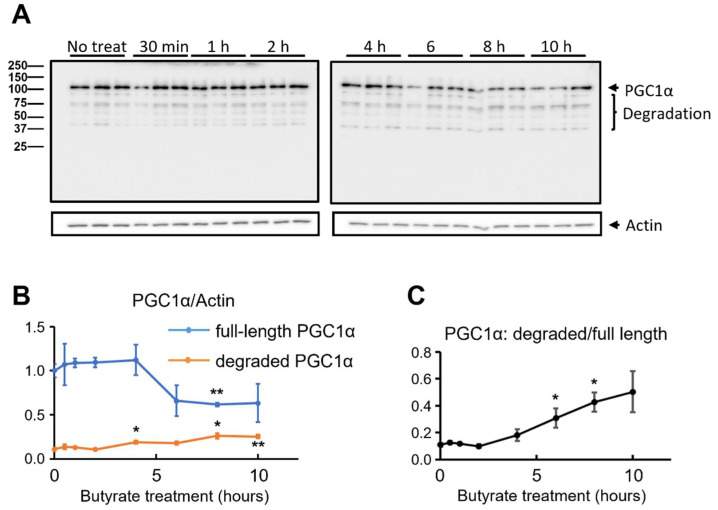
Time-dependent changes in the PGC1α protein level in NSC34-G93A cells following incubation with 1 mM butyrate. The 1 mM incubation was started 24 h after the transfection. The control groups received no butyrate treatment. (**A**) Protein samples were collected at 30 min and at 1, 2, 4, 6, 8, and 10 h post-treatment and analyzed via immunoblotting assay. (**B**) Time-dependent changes in both full-length (blue line) and degraded (orange line) PGC1α. (**C**) The ratio of degraded/full-length PGC1α. The data points are presented as the mean ± SE. *n* = 3 independent experiments. * *p* < 0.05; ** *p* < 0.01.

It has been reported that the intracellular concentration of PGC1α is tightly regulated by proteasome-mediated degradation [65]. Molinari et al. found that the potency of the transcription activator was inversely correlated with its cellular concentration and proposed that recruiting the transcription activator to DNA-bound receptor proteins greatly enhanced the degradation of the activator [66]. Thus, we carried out an analysis of the cellular PGC1α protein levels within the first 10 h of the butyrate incubation, starting as early as 30 min post-treatment. As shown in Figure 4A, the level of the full-length protein of PGC1α started to drop at 6 h post the butyrate addition. Importantly, we indeed observed the degraded bands in the immunoblotting. To better dissect the time-dependent changes in the PGC1α protein level, we first calculated the PGC1α/actin band intensity ratio for both full-length and degraded PGC1α at all time points. Then, all the data points were normalized to the value of the full-length PGC1α/actin ratio of the non-treated sample (at Time 0) and are presented in Figure 4B. A significant increase in degraded PGC1α was detected 4, 8, and 10 h post butyrate treatment, while the level of full-length PGC1α decreased significantly at 8 h of treatment. The ratio of degraded/full-length PGC1α was also calculated and is presented in Figure 4C, showing a significant increase at 6 and 8 h post the butyrate incubation.

### 3.4. Butyrate Treatment Increased the Expression of Key Molecules Involved in the Mitochondrial Electron Transport Chain of NSC34-G93A Cells

To investigate the downstream response of mitochondria to butyrate-mediated PGC1α signaling activation in NSC34-G93A cells, we examined both the mRNA and protein expression levels of the key molecules MTCO1, MTCO2, and COX4, which are critical for mitochondrial electron transportation [67,68,69]. One day after the transfection, the NSC34-G93A cells were incubated with 1 mM butyrate in the culture medium. The RNA samples collected at 4, 8, 24, and 48 h after butyrate incubation were used for RT-qPCR analysis. The protein samples collected at 1, 2, 3, and 4 days post the butyrate incubation were analyzed by immunoblotting assay.

**Figure 5:**
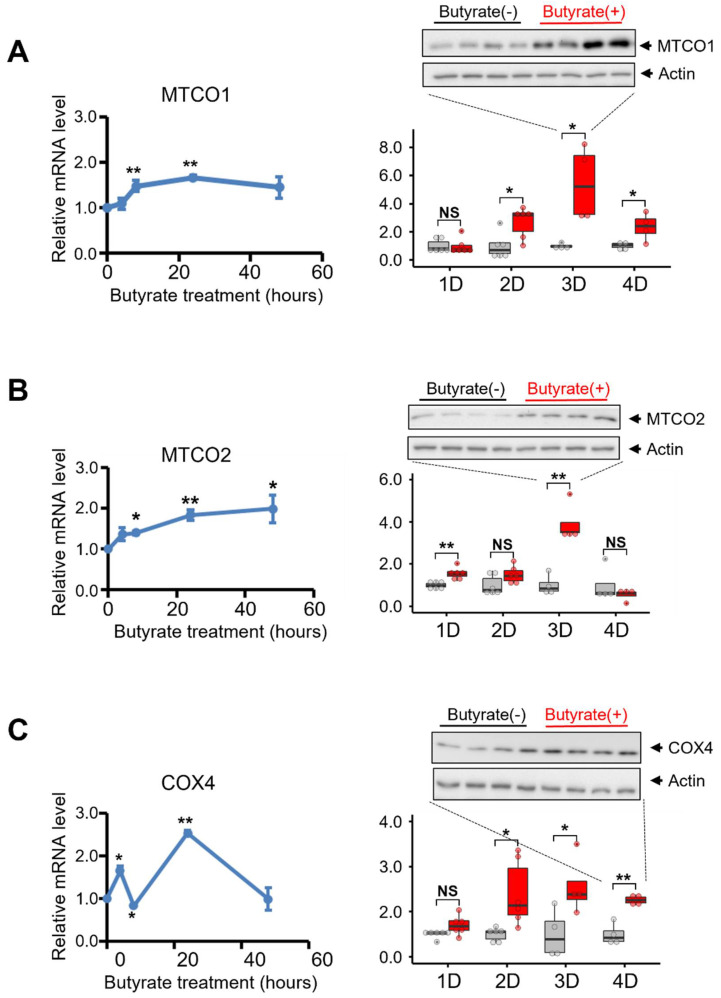
The effects of butyrate treatment on the expression of MTCO1, MTCO2, and COX4 in NSC34-G93A cells. NSC34 cells were transfected with mt-SOD1^G93A^-GFP. The incubation of 1 mM butyrate was started 24 h after the transfection. RNA was extracted after 4, 8, and 48 h post the butyrate treatment. (**A**–**C**, **left** panels): mRNA levels of MTCO1, MTCO2, and COX4 were determined by RT-qPCR and normalized to the levels in cells without the butyrate treatment. *n* = 3. (**A**–**C**, **right** panels): Relative protein levels of MTCO1, MTCO2, and COX4 with/without butyrate treatment from 1 to 4 days were analyzed using ImageJ with actin as a reference. Representative immunoblotting images are shown. *n* = 4. For the box and dot plot, the box bottom, median line, and box top represent the 25th (Q1), 50th (Q2), and 75th (Q3) percentiles, respectively. Whisker ends represent Q1 − 1.5*IQR and Q3 + 1.5*IQR, respectively. IQR is the interquartile range (Q3 − Q1). * *p* < 0.05; ** *p* < 0.01; NS: not significant.

The mRNA levels of NSC34-G93A cells without butyrate treatment were used as the baseline (Time 0) for calculating the time-dependent changes in the relative mRNA levels during the butyrate incubation (Figure 5A, left panels). The mRNA level of mitochondrion-encoded MTCO1 was increased by (1.5 ± 0.1)-fold 8 h after the butyrate addition. This elevated level was maintained for up to 48 h at the end of the experiment. At the protein level, MTCO1 increased by (2.7± 0.4)-fold at 2 days post the butyrate incubation, reaching (5.4 ± 1.3)-fold at 3 days post the butyrate incubation. The protein level of MTCO1 was still (2.4 ± 0.5)-fold over the control at 4 days after the butyrate incubation (Figure 5A, right panel).

The mitochondrion-encoded MTCO2 showed similar induction at the mRNA and protein levels. The mRNA level of MTCO2 showed fold changes of 1.4 ± 0.04, 1.8 ± 0.1, and 2.0 ± 0.3 at 8, 24, and 48 h, respectively, after the butyrate addition (Figure 5B, left panel). The protein level of MTCO2 started to show a significant increase one day after the butyrate incubation ((1.6 ± 0.1)-fold) and reached its peak on the third day at (3.9 ± 0.5)-fold. On the fourth day, no significant difference was detected between the treated and non-treated samples for the MTCO2 protein.

The mRNA level of nuclear-encoded COX4 was significantly enhanced by (1.6 ± 0.1)-fold at 4 h post the butyrate incubation, while at 8 h, the mRNA level of COX4 decreased slightly compared with the control (0.8 ± 0.01). Importantly, at 24 h post the butyrate incubation, the mRNA level of COX4 increased (2.5 ± 0.1)-fold compared with the control. At 48 h, the level dropped to the same level as that of the non-treated cells (1.0 ± 0.1) (Figure 5C, left panel). Similar to those of MTCO1 and MTCO2, the protein level of COX4 also significantly increased with fold changes of 2.2 ± 0.4, 3.1 ± 0.7, and 2.5 ± 0.1 at 2, 3, and 4 days post the butyrate incubation (Figure 5C, right panel). It is worth noting that the increased protein level of COX4 persisted even after the mRNA level reversed to the basal level.

## 4. Discussion

Previous studies showed that butyrate feeding improved neuromuscular function and slowed down the disease progression in an ALS mouse model with overexpression of a human ALS-associated mutation, hSOD1^G93A^ [1,2]. However, the molecular mechanisms underlying the beneficial effect of butyrate on motor neurons remain largely elusive. The NSC34-G93A cultured motor-neuron-like cells provide an opportunity for us to further examine the potential molecular bases underlying the beneficial role of butyrate at the cellular level.

Abnormal morphology of neuronal mitochondria has been found to be a common pathological denominator in both SOD1 and TDP43 ALS mouse models [70]. Our previous studies in skeletal muscle and bone cells also showed that a defective mitochondrial network with enhanced mitochondrial fragmentation is implicated in both muscle wasting and bone degeneration in the ALS SOD1^G93A^ mouse model [51,54]. A study from Raimondi et al. reported that NSC34 cells with overexpression of hSOD1^G93A^ exhibit increased mitochondrial fragmentation [53]. Similarly, studies from Magrane et al. also demonstrated similar morphology changes when mutant hSOD1 was targeted to the mitochondrial intermembrane space in NSC34 cells [52]. In line with published studies, the NSC34-G93A cells demonstrated a similar phenotype, with ~44% of the cell population showing fragmented mitochondria. Importantly, the application of 1 mM butyrate in the culture medium for 2 to 3 days increased the cell population of networked mitochondria up to threefold (Figure 1D). This apparent morphological change in the mitochondrial network does not seem to involve noticeable changes in the total mitochondrial volume (Figure 1E). However, a trend of increase in mitochondrial DNA content was detected on the first day following the butyrate incubation. It is likely that one of the early responses of NSC34-G93A cells to the butyrate treatment is enhanced mitochondrial fusion to form a more functionally connected mitochondrial network without detectable changes in total mitochondrial volume.

It is known that mitochondrial fusion is required for efficient respiratory capacity by spreading metabolites, enzymes, and counteracting mtDNA mutations throughout the mitochondrial network [71]. The increased mitochondrial networking could imply improved mitochondrial bioenergetics. We therefore compared the mitochondrial metabolism status of NSC34-G93A cells in the presence or absence of butyrate treatment using Seahorse technology to determine the oxygen consumption rate of mitochondria (Figure 2). We found no significant difference in basal respiration or the ATP production of mitochondria in the non-stress state after 48 h of the butyrate treatment. These data may suggest that butyrate does not affect mitochondrial energy production in the basal condition. However, cells treated with butyrate displayed significantly higher maximal respiration capacity. Importantly, the spare respiratory capacity was increased by 4.7-fold following the butyrate treatment, suggesting that butyrate treatment improved mitochondrial bioenergetics.

As PGC1α is a key regulator of energy metabolism by remodeling the cell toward more oxidative metabolism [72], we investigated whether PGC1α in NSC34-G93A cells was involved in the response to the butyrate treatment. As shown in Figure 3A, the mRNA level of PGC1α was induced as early as 4 h following the butyrate treatment. The mRNA level reached its peak with an increase of ~19-fold at 8 to 24 h and was maintained as high as ~8-fold after 48 h of the butyrate treatment. Unexpectedly, the protein expression levels of PGC1α were not consistent with the increased mRNA levels at 8, 24, and 48 h post-treatment. Instead, we observed a significantly reduced protein level at 8 and 24 h post-treatment (Figure 3B,C). Published studies showed that rodents with long-term butyrate feeding had increased levels in both mRNA and protein expression of PGC1α in brown adipose tissue and skeletal muscle [73,74]. Tissue differences or treatment duration may account for the different responses to butyrate. It is also possible that the degradation rate of PGC1α is faster than its synthesis in NSC34-G93A cells following a short-term butyrate treatment. Pettersson-Klein et al. reported that PGC1α has a half-life within 1 h [75]. As suggested by Sano et al. the short half-life of PGC1α may best fit its fine-controlled rapid response to cellular stress [76]. Published studies also revealed that the degradation rates of transcription factors in the nucleus are positively correlated to their functions [66]. In other words, the butyrate-induced activation of PGC1α protein could lead to its faster degradation inside the nucleus, considering its short half-life of 30 min. We therefore examined the protein expression levels of PGC1α in a more extended time window, starting as early as 30 min and covering 1, 2, 4, 6 and 10 h post butyrate application (Figure 4). However, there was still no increase detected in the protein level of full-length PGC1α as early as 30 min following the treatment. Instead, the protein degradation started to show as early as 6 h post treatment. It was still possible that we missed the time windows (shorter than 30 min or longer than 48 h) when the protein levels of PGC1α could be increased by the butyrate treatment. Although this is an unexpected result, we believe that this extensive data set has value to be reported to the research field, and future studies are deserved to further explore this interesting phenomenon.

Although an increased protein level of PGC1α was not detected, the butyrate treatment led to a drastic induction of the transcription of PGC1α. Considering the enhanced mitochondrial respiration capacity, we speculated that the butyrate treatment enhanced mitochondrial bioenergetics via PGC1α activation. To further test our hypothesis, we examined the key molecules (MTCO1, MTCO2, and COX4) involved in mitochondrial oxidative phosphorylation regulated downstream by PGC1α. Cytochrome c oxidase (COX) is the key component of the mitochondrial electron transport chain [77,78]. Increased activity of COX leads to higher levels of ATP production [77]. Mammalian COX contains 13 subunits, 3 of which are encoded by mitochondrial DNA (i.e., MTCO1–3), and the rest are encoded by nuclear DNA (i.e., COX4) [77]. We examined the time-dependent changes in both the mRNA and protein expression levels of MTCO1, MTCO2, and COX4 following the butyrate treatment (Figure 5). In line with the increased mRNA level of PGC1α, the mRNA levels of MTCO1 and MTCO2 increased at 8 h and peaked at 24 h following the butyrate treatment. A significant increase in COX4 mRNA was detected as early as 4 h post the butyrate incubation. Interestingly, the mRNA level of COX4 slightly dropped below the baseline at 8 h post treatment. We have no proof about the nature of this small reduction. However, similarly to those of MTCO1 and MTCO2, the COX4 mRNA level peaked at 24 h post treatment. Importantly, the protein expression of MTCO1, MTCO2, and COX4 increased significantly, peaking at 3 days following the butyrate treatment. This set of data further supports our hypothesis that induction of the transcription of PGC1α likely leads to downstream changes in genes and proteins in NSC34-G93A cells that favor mitochondrial bioenergetics.

Induction of PGC1α transcription is generally considered to play a central role in mitochondrial biogenesis [79,80]. The drastic elevation in PGC1α transcription suggests that butyrate treatment may activate mitochondrial biogenesis in NSC34-G93A cells. Mitochondrial biogenesis is a complex process that includes, but is not limited to, induction of PGC1α mRNA/protein levels; nuclear translocation of PGC1α; downstream or upstream signaling molecules of PGC1α; mRNA, protein, or enzymatic activity of mitochondrial components; increased mitochondrial DNA copy numbers and mitochondrial density, etc. Not all the features of typical mitochondrial biogenesis were observed in our current study following the butyrate treatment. However, we indeed demonstrated a drastic and time-dependent induction of PGC1α transcription in response to the butyrate treatment, along with a persistent increase in both the mRNA and protein levels of key molecules involved in mitochondrial electron transport chain, which are encoded by both the nuclear and mitochondrial genome. Most importantly, in line with our observations at the mRNA and protein levels, the mitochondrial functional assessment by Seahorse revealed an enhanced capacity for oxidative phosphorylation that was further supported by the improved mitochondrial network with a significant increase in mtDND/nDNA following the butyrate incubation. However, an increase in mitochondrial volume during the first three days of butyrate treatment was not detected, and the PGC1α protein expression level was also paradoxically reduced. It is possible that butyrate-induced mitochondrial biogenesis might be initiated but not fully established within 2 or 3 days of treatment in an in vitro cellular model. Future studies using in vivo ALS animal models should be employed to further characterize this possibility.

It is worth noting the shortcoming of the NSC34 cell culture [81] as an ALS cellular model, due to its neuroblastoma linage, in which the oncogene N-myc could impact diverse cellular responses and set potential limitations on studying the mechanisms of motor neuron death. While all current available cellular models for ALS have their own shortcomings, future studies are needed to include other alternative ALS cellular models and to correlate the results with in vivo animal model tests. While our initial characterization of the molecular nature underpinning the butyrate effect was made with the concentration of 1 mM on NSC34-G93A cells, future studies should ensure a dose-dependent toxicity evaluation on multiple cellular and animal models of ALS. Additionally, there are transporters and receptors involved in the cellular responses to short-chain fatty acids, including butyrate [82,83,84,85], so identifying specific transporters or receptors for butyrate in neuronal cells could add another layer to understanding the effect of butyrate as a potential therapy for ALS. Butyrate has been extensively studied as an HDAC inhibitor, but its multiple cellular effects have also emerged as a ligand for a subset of G protein-coupled receptors and as a direct substrate for mitochondrial energy metabolism [40], which could broadly affect the disparate cellular functions. Future studies are required to further explore the molecular nature of the potential multifaceted effects of butyrate in preserving neuromuscular function for the treatment of ALS.

## 5. Conclusions

Combining live cell imaging, Seahorse mitochondrial respiratory function assessment, and the quantification of time-dependent mRNA and protein levels in NSC34-G93A cells, we revealed significantly enhanced spare respiratory capacity following two days of butyrate incubation. This butyrate-induced functional improvement was further supported by the enhanced mitochondrial network and the increased mRNA and protein levels of essential components of the mitochondrial electron transport chain (MTCO1, MTCO2, and COX4), which are downstream molecules regulated by PGC1α, a master regulator of mitochondrial biogenesis. Importantly, we also observed an early burst of PGC1α transcription, indicating that activation of the PGC1α signaling axis could underline the beneficial effects of butyrate in improving mitochondrial bioenergetics in NSC34-G93A cells. Although the total mitochondrial volume showed no detectable increase, the mitochondrial DNA appeared to increase after 24 h of butyrate incubation. We speculate that mitochondrial biogenesis is initiated by butyrate but not fully established in the time window tested in this in vitro cellular model. Future studies using ALS animal models should be conducted to further characterize this possibility.

## Figures and Tables

**Table 1:** Primers used in qPCR.

Primer	Sequence
ACTb_F	ACTGTCGAGTCGCGTCCACC
ACTb_R	CACCATCACACCCTGGTGCC
MTCO1_F	ACTCATCCCTTGACATCGTGCT
MTCO1_R	GCGAAGTGGGCTTTTGCTCA
MTCO2_F	CTACAAGACGCCACATCCCCT
MTCO2_R	ATGCGTAGAGAGGGGAGAGCA
COX4_F14	CTGCCCGGAGTCTGGTAATG
COX4_R122	CAGTCAACGTAGGGGGTCATC
PGC1a_F32	TATGGAGTGACATAGAGTGTGCT
PGC1a_R165	CCACTTCAATCCACCCAGAAAG
ND2_F	GTCACACAAGCAACAGCCTCA
ND2_R	TCAGAAGTGGAATGGGGCGAG
HK2_F	GCCAGCCTCTCCTGATTTTAGTGT
HK2_R	GGGAACACAAAAGACCTCTTCTGG

## Data Availability

All the data are available within the article.
